# Morphological and molecular analyses of a *Meloidogyne mali* population with high intragenomic rRNA polymorphism

**DOI:** 10.21307/jofnem-2020-105

**Published:** 2020-11-06

**Authors:** Jianfeng Gu, Yiwu Fang, Lele Liu

**Affiliations:** Ningbo Customs Technical Centre (Ningbo Inspection and Quarantine Science Technology Academy), 8 Huikang Road, Ningbo, 315100, Zhejiang, P.R. China

**Keywords:** *Acer palmatum*, Haplotype, Japanese maple, Mitochondrial COI, Phylogeny, Root-knot nematode

## Abstract

Root-knot nematode, *Meloidogyne mali* can cause damage in trees, shrubs, and herbaceous plants, and was placed on the EPPO Alert List in 2014. In the present study, we report a population isolated from Japanese maple. The recovered population is described by detailed morphological and molecular approaches. The molecular phylogentic analysis based on 28S rRNA, ITS, and mitochondrial COI genes places the population in the clade together with other *M. mali* sequences available in GenBank. The cloned sequences of the 28S rRNA gene revealed a high intragenomic rRNA polymorphism where the polymorphic copies are spread across *M. mali* clade. Similarly, we also found high variation in the mitochondrial COI gene. Among four haplotypes in *M. mali*, three occur in the newly found population. Our study provides the first report of intragenomic polymorphism in *M. mali*, and the results suggest that intragenomic polymorphism maybe widespread in *Meloidogyne.*

*Meloidogyne mali* (Itoh et al., 1969) is a root-knot nematode (RKN) causing significant damage by inducing root galls on its host plant and consequently reduced host growth by interfering with the uptake of water and nutrients. *M. mali* has a wide host range, typically on trees, but can also parasitize on shrubs and herbaceous plants (Ahmed et al., 2013).

*M. mali* was first described in Japan in 1969, with the type host apple (*Malus domestica* Borkh.) (Itoh et al., 1969). In 2000, a root-knot nematode parasitizing elm (*Ulmus chenmoui* W.C. Cheng) was described as a new species, *M. ulmi* (Palmisano and Ambrogioni, 2000); however, it was later synonymized with *M. mali* (Ahmed et al., 2013). Currently, *M. mali* has been reported worldwide from Japan, France, Italy, the Netherlands (Karssen et al., 2008), and more recently in the United States (Eisenback et al., 2016) and the United Kingdom (Prior et al., 2019). In China, although *M. mali* was reported in citrus in Fujian province (Zhang and Weng, 1991) and populus in Henan province (Li and Yu, 1991), both descriptions lack detailed morphological or molecular data, and neither citrus nor populus are known to be the host of *M. mali*. Thus, the presence of *M. mali* in China remains questionable, as does the suitability of citrus and populus as hosts. Due to its wide host and broad distribution, *M. mali* was placed on the EPPO Alert List in 2014.

Intragenomic polymorphisms have been found in various plant–parasitic nematode taxa, including genera *Cephalenchus*, *Pratylenchus*, *Rotylenchulus*, and *Xiphinema* (Pereira and Baldwin, 2016; Van Den Berg et al., 2016; Qing et al., 2020b). In *Meloidogyne*, the intragenomic polymorphisms have been noticed in *M. incognita* (Kofoid and White, 1919; Chitwood, 1949), *M. arenaria* (Neal, 1889; Chitwood, 1949), and *M. javanica* (Treub, 1885; Chitwood, 1949), which have reticulated evolution background (Georgi and Abbott, 1998; Lunt, 2008). However, this character is less known in the other *Meloidogyne* groups, especially those in the basal clades. In the present study, we reported that a *M. mali* population was imported from Japan in Ningbo Port, China, with high intragenomic rRNA polymorphisms. We describe this population by detailed morphology and molecular phylogeny, and compare it with other populations in GenBank by mitochondrial COI haplotype network analysis.

## Materials and methods

### Isolation and morphological observation of nematodes

Roots and a little soil and rhizosphere medium associated with maple (*Acer palmatum* Thunb f.) imported from Japan to Ningbo, China, were collected in a plastic bag. The symptoms of the trees were not observed. Nematodes were extracted in the laboratory by a modified Baermann funnel technique for 24 hrs. When second-stage juveniles and males of *Meloidogyne* spp. were detected, the remaining roots were studied and observed with a stereomicroscope, and females were dissected directly from galled roots. Specimens were prepared for light microscopy (LM); males and J2s were relaxed with gentle heat, fixed in a solution of 4% formaldehyde, and processed using the glycerin-ethanol method (Seinhorst, 1959). Perineal patterns of mature females were prepared as described by Hartman and Sasser (1985). The perineal pattern was trimmed and transferred to a drop of glycerin for observation. Nematodes were measured and photographed with a Zeiss Imager Z1 microscope equipped with a Zeiss AxioCam MRm CCD camera.

### Molecular analyses

For extracting DNA, a single juvenile was handpicked, individually examined on a temporary slide, photographed, and transferred to a small drop of worm lysis buffer (WLB: 20 mM Tris-HCl, pH 8.0, 100 mM KCl, 3.0 mM MgCl_2_, 2.0 mM DTT, and 0.9% Tween20) following the protocol used by Li et al. (2008). Seven juveniles were prepared for DNA analysis separately; when examined under the microscope, there were both long and short individuals. The ITS1/2 region was amplified with the primer V1 (Vrain, 1993) and 5.8S (Cherry et al., 1997), the partial 18S region was amplified with SSU-F-04 and SSU-R-81 (Griffiths et al., 2006), the D2-D3 region of 28S was amplified with D2A/D3B (De Ley et al., 1999) and the COI mtDNA region was amplified with the primer JB3 and JB5 (DeRycke et al., 2005). PCR reactions were done following the protocol of Ye et al. (2007) and Li et al. (2008). Amplification success and amplicon size were verified in 1.0% agarose gel stained with ethidium bromide. All the positive PCR products were sent for direct sequencing first. For COI, the results were good, but for ITS1/2 and 28S, the chromatogram was not interpretable. So, positive ITS1/2 and 28S PCR products were cloned with the pMD 18-T Vector cloning Kit (Promega) using TOP10-competent cells following the manufacturer’s instructions before sequencing. PCR products were sequenced in both directions with PCR primers in Invitrogen, Shanghai, China. The acquired sequences were deposited in the GenBank with accession numbers MT407598-MT407600 for COI, MT406751-MT406767 for 28S, and MT397000-MT397002 for ITS1/2.

### Phylogenetic analyses

The obtained sequences were analyzed with other relevant *Meloidogyne* sequences available in curated plant-parasitic nematode database PPNID (Qing et al., 2020a). Multiple alignments of the different genes were made using the E-INS-i algorithm of MAFFT v. 7.205 (Katoh and Standley, 2013). The best-fitting substitution model was estimated using AIC in jModelTest v. 2.1.2 (Darriba et al., 2012). Maximum likelihood (ML) and Bayesian (BI) analysis were performed at the CIPRES Science Gateway (Miller et al., 2010), using RA x ML 8.1.11 (Stamatakis et al., 2008) and MrBayes 3.2.3 (Ronquist and Huelsenbeck, 2003), respectively. ML analysis included 1,000 bootstrap (BS) replicates under the GTRCAT model. Bayesian phylogenetic analysis was carried out using the GTR + I  +G model for 1 × 10^7^ generations, and Markov chains were sampled every 100 generations and 25% of the converged runs were regarded as burn-in. Gaps were treated as missing data for all phylogenetic analyses. The TCS networks of mitochondrial COI haplotype were implied in the PopART program.

## Results

### Morphological characteristics

(Figs. 1, 2)

**Figure 1:**
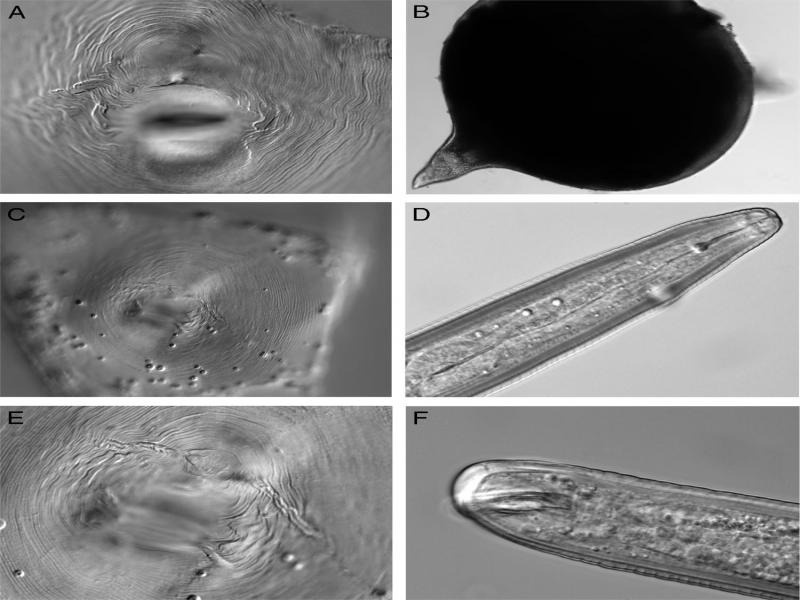
Light micrographs of females and males of *Meloidogyne mali*. A, C, E: perineal pattern; B: whole female; D: male head; E: male tail (scale bars = 10 μm).

**Figure 2:**
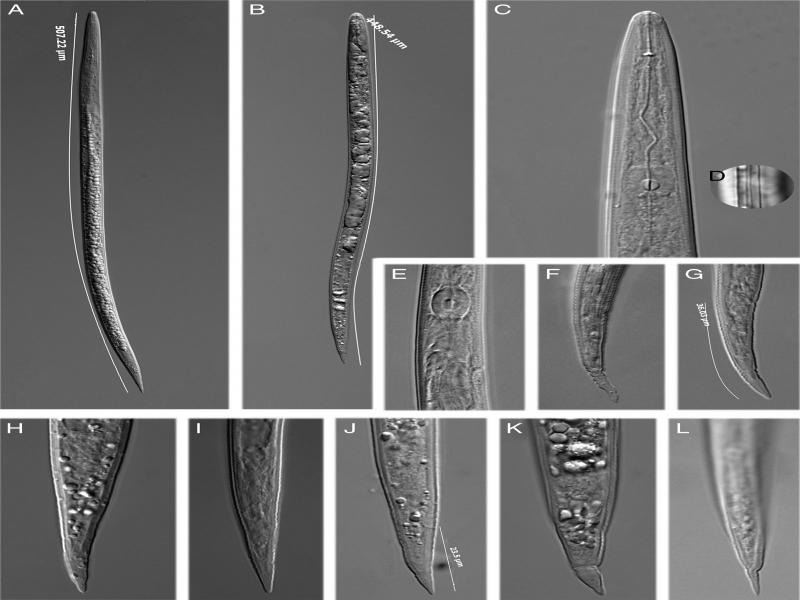
Light micrographs of juveniles of *Meloidogyne mali*. A: whole body of a long juvenile; B: whole body of a normal juvenile; C: anterior region; D: lateral lines; E: median bulb region; F-L: tail region. (scale bars = 10 μm).

#### Female (*n* = 3)

Body length (including neck) = 710 ± 32.7(670-750) µm, neck = 90.0 ± 9.4(78.0-101.0) µm, stylet = 12.0 ± 1.1(10.9-13.4) µm, dorsal pharyngeal grand orifice to stylet base = 3.5 ± 0.7(2.6-4.4) µm, vulval slit length = 20.7 ± 2.1 (18.3-23.5) µm, vulval slit to anus = 18.6 ± 2.1(15.6-20.3) µm, and interphasmidial distance = 21.4 ± 1.6(19.6-23.4) µm.

Mature females were completely enclosed in the root tissue, body white or slightly darker, usually spherical or onion-shaped with a short neck. Neck tapering at the anterior end, less than one-fourth of body length. The head region was small, slightly set off from the body. The cuticle was comparatively thick. The excretory pore was generally located slightly posterior to the dorsal pharyngeal gland orifice. The stylet was slender, short, and slightly backwardly sloping to the anterior concave. The general shape of the perineal pattern which have been studied ranged from low, rounded, to oval, made up of smooth striae finely spaced. The dorsal arch was mostly low and rounded. A lateral field with weak lateral lines, but the number is hard to identify, maybe two.

#### Male (*n* = 1)

Body length = 1,365 µm, width = 40 µm, *a* = 34.1, *b* = 14.5 (total length divided by the distance from the anterior end to the middle of the valve plate in median bulb), stylet = 19.7 µm, dorsal pharyngeal gland orifice to stylet base = 8.9 µm, excretory pore to the anterior end = 156.4 µm, spicules = 34.9 µm, and gubernaculum = 9.6 µm.

The body was long, slender, tapering rather at both head and tail portions, and being narrower anteriorly. The body was almost straight when killed by gentle heat showing a twist in the posterior portion. The lateral field was about one-third the body width with four incisures. The excretory pore was located 21 annules behind the median esophageal bulb. The head region was not clearly set off from the body. The stylet with rounded basal knobs was also described.

#### Second-stage juvenile (*n* = 20) (morphometrics, see Table 1)

The body was slender, tapering at both the head and tail portions and cuticular annulation was fine but fairly distinct. The lateral field was more than one-third of body width, with four incisures. The head was slightly set off from the body, with a thin labial cap. The stylet was delicate, with small knobs sloping backward. The excretory pore was often difficult to observe. The hemizonid measured 74.4 to 85.5 µm from the head. The tail was conoid and short tapering to a finely rounded or slightly pointed terminus, sometimes more or less rounded. Cuticular constrictions were sometimes present. Hyline tail terminus was variable in length, measuring 6.8 to 12.0 µm.

**Table 1.** Morphometrics of the described *M. mali* population from Japan compares to a population detected in 2013.

	*M. mali* population with high intragenomic rRNA polymorphism	*M. mali* population (Gu et al., 2013)
*n*	20	20
Body length	445 ± 28.3 (401-507)	425 ± 30.1 (362-466)
Body width at mid-body	14.0 ± 0.6 (13.1-15.4)	14.0 ± 1.1 (13.1-18.1)
Anterior end to median bulb valve	52.7 ± 4.1 (46.8-58.7)	53.5 ± 2.1 (49.5-56.8)
Anterior end to the hemizonid	79.0 ± 3.8 (74.4-85.5)	–
Anterior end to the excretory pore	75.1 ± 4.2 (72.8-83.3)	74.1 ± 4.2 (68.8-82.3)
Head width	5.8 ± 0.3 (5.3-6.4)	5.1 ± 0.41 (4.1-5.7)
Head height	3.0 ± 0.2 (2.6-3.4)	2.6 ± 0.32 (2.1-3.2)
Stylet length	10.4 ± 0.5 (9.4-11.1)	10.5 ± 0.5 (9.5-11.6)
Stylet cone length	5.4 ± 0.2 (5.1-5.8)	5.7 ± 0.5 (4.7-6.7)
Stylet knob height	1.5 ± 0.4 (1.0-2.6)	1.2 ± 0.2 (0.8-1.6)
Stylet knob width	2.2 ± 0.3 (1.8-2.8)	2.1 ± 0.2 (1.8-2.7)
DGO	3.2 ± 0.5 (2.4-4.0)	4.4 ± 0.57 (3.5-5.5)
Tail length	30.5 ± 4.5 (23.5-35.8)	32.7 ± 3.0 (29.2-39.3)
Body width at the anus	7.8 ± 0.7 (6.1-8.7)	7.9 ± 0.9 (5.9-9.6)
Hyaline tail terminus length	9.8 ± 1.6 (6.8-12.0)	7.2 ± 2.3 (3.9-9.3)
*a*	31.9 ± 2.5 (27.4-36.6)	30.4 ± 2.6 (25.2-34.5)
*b*′	–	8.0 ± 0.6 (6.8-9.2)
*c*	14.8 ± 2.5 (12.1-19.4)	13.2 ± 1.1 (11.6-15.3)
*c*′	3.8 ± 0.6 (3.0-4.8)	4.2 ± 0.7 (3.1-5.6)
*m*	52.4 ± 2.4 (49.5-58.6)	54.5 ± 3.8 (47-60.3)
*O*	30.6 ± 4.7 (22.9-38.8)	41.9 ± 5.8 (31.6-53.9)
Head width/head height	1.9 ± 0.1 (1.7-2.2)	2.0 ± 0.2 (1.6-2.3)
H%	33.2 ± 8.6 (19.1-47.3)	21.7 ± 5.9 (12.7-27.8)

**Note:** All measurements are in µm and in the form: mean ± s.d. (range).

#### Diagnosis and relationships

Compared to the original description, the morphological characteristics, especially the female perineal patterns and the juvenile tail shape are similar.

But for juvenile morphometrics, small differences exist: some individuals of our population had a much longer body length, up to 507 µm, while in reported isolates, the longest one was only 466 µm (Gu et al., 2013). Furthermore, some of such long individuals show a very short tail length, only about 25 µm (31-33 µm on average). This characteristic is also expressed by the c value (the highest c value is 19.4, while the former isolate values are only 15.3 (Gu et al., 2013) and 17.4 (Ahmed, 2013)).

## Molecular profiles and phylogenetic status

**Figure 3:**
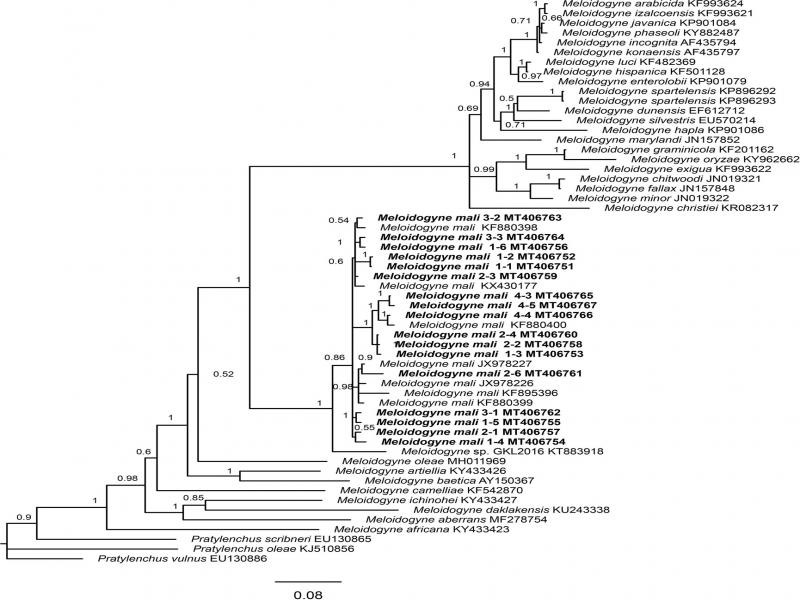
Phylogenetic relationships within the genus *Meloidogyne* as inferred from Bayesian analysis of the D2-D3 region of the 28S rRNA gene sequences using the GTR + I + G model. Posterior probabilities are given in clade nodes. Newly obtained sequences are indicated in bold and the sequence codes are given in specimen-clone.

**Figure 4:**
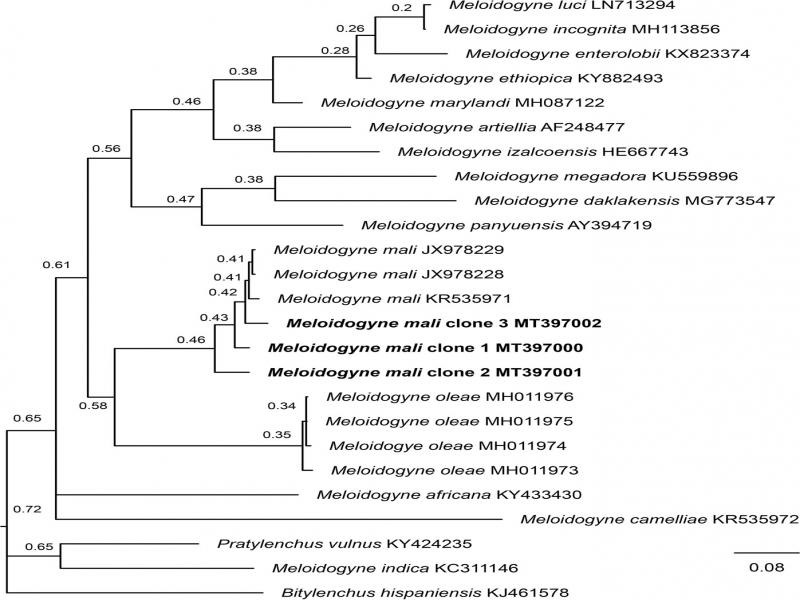
Phylogenetic relationships within the genus *Meloidogyne* as inferred from Bayesian analysis of the ITS sequences using the GTR + I + G model. Posterior probabilities are given in clade nodes. Newly obtained sequences are indicated in bold.

**Figure 5:**
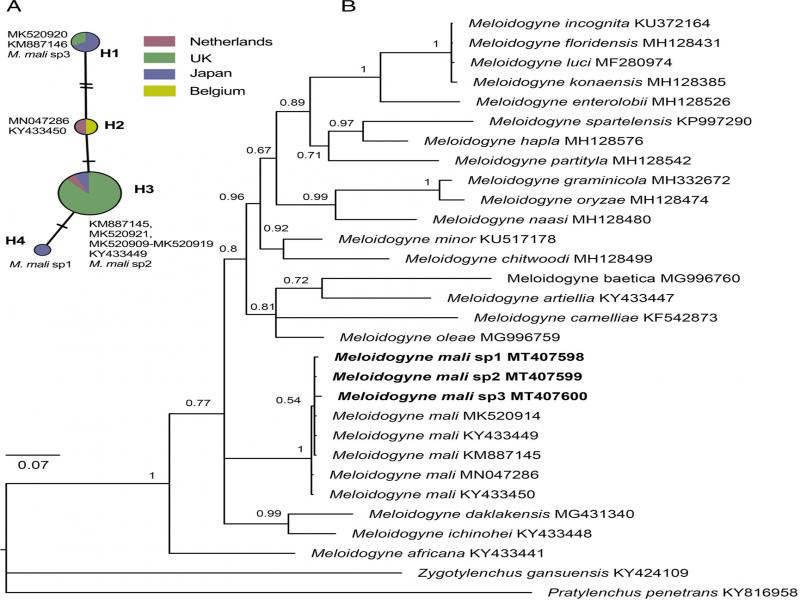
The TCS haplotype networks of *Meloidogyne mali* (A) and the Bayesian majority rule consensus tree of Meloidogyne mali based on mitochondrial COI gene (B). A: Pie chart indicates the composition and proportion of haplotypes in each location. Each circle corresponds to one haplotype and its size is proportional to its frequency. Each line connecting the haplotypes refers to a mutational step. Marks on the lines indicate the number of steps. B: Values at branche nodes are Bayesian posterior probabilities. Newly submitted sequences are indicated in bold.

The acquired COI phylogeny concurred with previous studies (Janssen et al., 2017; Álvarez-Ortega et al., 2019) in general topologies and supportive values. In all analyses, our population clustered with other *M. mali* sequences available in GenBank. The monophyletic *M. mali* was fully supported in mitochondrial COI gene (PP = 1), but only moderately (PP = 86) and weakly supported (PP = 46) in 28S and ITS, respectively. In 28S phylogenetic tree, *M. mali* was sister to an unidentified Chinese *Meloidogyne* population forming a fully supported clade (PP = 1), while in ITS, it was sister to *M. oleae* Archidona-Yuste, Cantalapiedra-Navarrete, Liébanas, Rapoport, Castillo and Palomares-Rius, 2018 in a weakly supported clade (PP = 0.58) and showed an unresolved mitochondrial COI phylogenetic tree (Figs. 3-5).

A total of 17 clones from four specimens were sequenced for 28S rRNA, and analysis of acquired sequences showed an overall p-distance of 0.032, with 87 variable sites and 39 singletons in an alignment of 798 nucleotides. The phylogeny analysis suggested that these clones were generally placed across *M. mali* clade, except for specimen 4 clustered together with a GenBank *M. mali* sequence (KF880400). Similarly, ITS also shows high rRNA variation where three clones were different and intragenomic polymorphisms are higher than the variation among populations. Compared with 28S rRNA, COI sequences in *M. mali* are more homogeneous. The haplotype network analysis recovered a total of four haplotypes among 21 sequences originated from the Netherlands, United Kingdom, Belgium, and Japan. The haplotype H3 is most common with 15 sequences, while haplotype H4 has been found only once. Remarkably, the three newly sequenced specimens belong to three different haplotypes, suggesting that our recovered population has a high haplotype diversity.

## Discussion

The identification and detection of *M. mali* are relatively difficult, given the long asymptomatic incubation period in host trees. The tree decline was only discriminative after several years when a high population density built up.

From a taxonomic aspect, less morphology and molecular data are available compared with tropical root-knot nematode (e.g., *M. incognita*, *M. javanica*, and *M. arenaria*). Practically, preparation of female perineal pattern is challenging. Regardless of the high intraspecific variation, our findings suggest that all these variations were placed in a monophyletic clade. Therefore, the rRNA-based molecular barcoding still remains a powerful tool for *M. mali* delimitation.

The rRNA polymorphisms are widespread across Nematoda (Pereira and Baldwin, 2016; Van Den Berg et al., 2016; Qing et al., 2020b). Current nematode taxonomy and phylogeny rely on rRNA genes (e.g., Holterman et al., 2006; Bert et al., 2008); thus, the presence of intragenomic rRNA variation may increase uncertainty in species identification and delimitation. For root-knot nematode, high rRNA variation has been noticed in the *M. incognita* genome (Qing et al., 2020b). This is not surprising as interspecific hybridization has been involved in the origin of tropical root-knot nematodes, and has played a role in shaping the patterns of their genetic diversity (Lunt, 2008). Different from more derived tropical root-knot nematodes, *M. mali* is close to the basal branch of *Meloidogyne* and likely to represent the ancestral state of the genus with several intermediate characteristics similar to those of *Pratylenchus* (e.g., with an incompletely swollen body with a protuberance, the spermatheca comprises a limited number of cells, with not-interlaced and not-lobe-like cells) (Janssen et al., 2017). Moreover, the presence of such characteristics in *M. mali* suggests that rRNA polymorphism is widespread across the genus *Meloidogyne* rather than parthenogenesis. Interestingly, rRNA polymorphism has also been reported from *Pratylenchus* (Qing et al., 2020b).

In an examination of our *M. mali* population (Gu et al., 2013; Gu and He, 2015) using the sequence chromatography method by Nadler et al. (2003) (polymorphisms were recognized in chromatography when both alternative nucleotide peaks were present in all sequence reactions representing both DNA strands, and when the minor nucleotide peak represented at least 25% of the major peak), we failed to recover polymorphism in all sequenced individuals. Although this is not confirmed by cloning analysis, this may indicate that rRNA polymorphism in *M. mali* is population-specific. The variation may be caused by a high level of intraspecific hybridization background, or the difference in reproduction strategies, as *M. mali*, was found to reproduce by amphimixis in the presence of males, but it remains possible to reproduce in meiotic parthenogenesis (Janssen et al., 2017). Unfortunately, our limited specimens did not allow us for a detailed examination of spermatheca and for further cloning.

*Meloidogyne* species are spread in long distances mainly through plants and seedling transportation. In recent years, *M. mali* has been repeatedly detected in imported plants from Japan. So living plants and seedlings with roots are possible to carry dangerous nematodes, and quarantine-treatment measurements, like chemical treatment, are impossible to kill all the nematodes in the roots. Quick detection and identification methods are required for national distribution research and port quarantine and inspection.
